# The Future of Medical Education

**Published:** 1916-02-26

**Authors:** 


### THE FUTURE OF MEDICAL EDUCATION.

In spite of the pressing occupations of the moment, not a few minds are beginning to contemplate the position which will exist at the close of the war and the special demands which this will bring with it. Neither the date nor the exact circumstances can be defined with confidence. But the event is certain, and at least some of the problems can be foreseen. Unless steps are taken in advance to solve these problems, or at least to prepare for their solution, their arrival will find us unready, and muddle and haste and blundering will be the order of the day. Schemes of imperial politics, trade organisation, fiscal arrangements, and other interests are already contemplating various developments, and there is a universal conviction that preparations must be made for a new world and a new order. So far as can be gathered from the public prints, the possibility of fresh educational movements, though vaguely recognised, has not yet called into being any practical measures or organised deliberations. Certainly this is true of medical education. Yet not less certainly is it true that the return of peace will mean the necessity for important modifications and for new enterprises if the necessities of the position are to be adequately met.

We are far from wishing to disparage the importance of the work of war committees and the like when we say that some of the "elder brethren" of the profession might usefully be spared from these labours in order that they might address their abilities to an organised study of the educational future. Doubtless particular institutions—universities and colleges—must in some measure be contemplating the situation, for it is forced on them by imperative financial considerations. But each of these necessarily has to consider its individual interest. What is wanted is a broader survey and a wider outlook, so that the whole question may be regarded from a national, or indeed from an imperial standpoint. This is the standpoint from which many things formerly having but a mere parochial significance will have to be looked at in the immediate future, and medical education, if it is to occupy a worthy position in the nation and in the Empire, must be treated in this spirit.

The first demand is the collection of facts. These are readily accessible, but there is urgent need for their ordered presentation and comparison. Especially is it necessary to study the extent of our educational resources and to inquire whether these are utilised wisely and economically. For many reasons, urgent at the moment, the question of research is just now very prominent, but really efficient teaching as a permanent demand is not less important, and efforts to promote this would be well repaid. To work to these ends a committee of inquiry is, of course, essential, and no body seems more suited for the initial steps than the Royal Society of Medicine. We have always expressed confidence in the abilities of the Society to serve the profession over a wide platform, and here is an opportunity to prove both its capacity and its public spirit.

